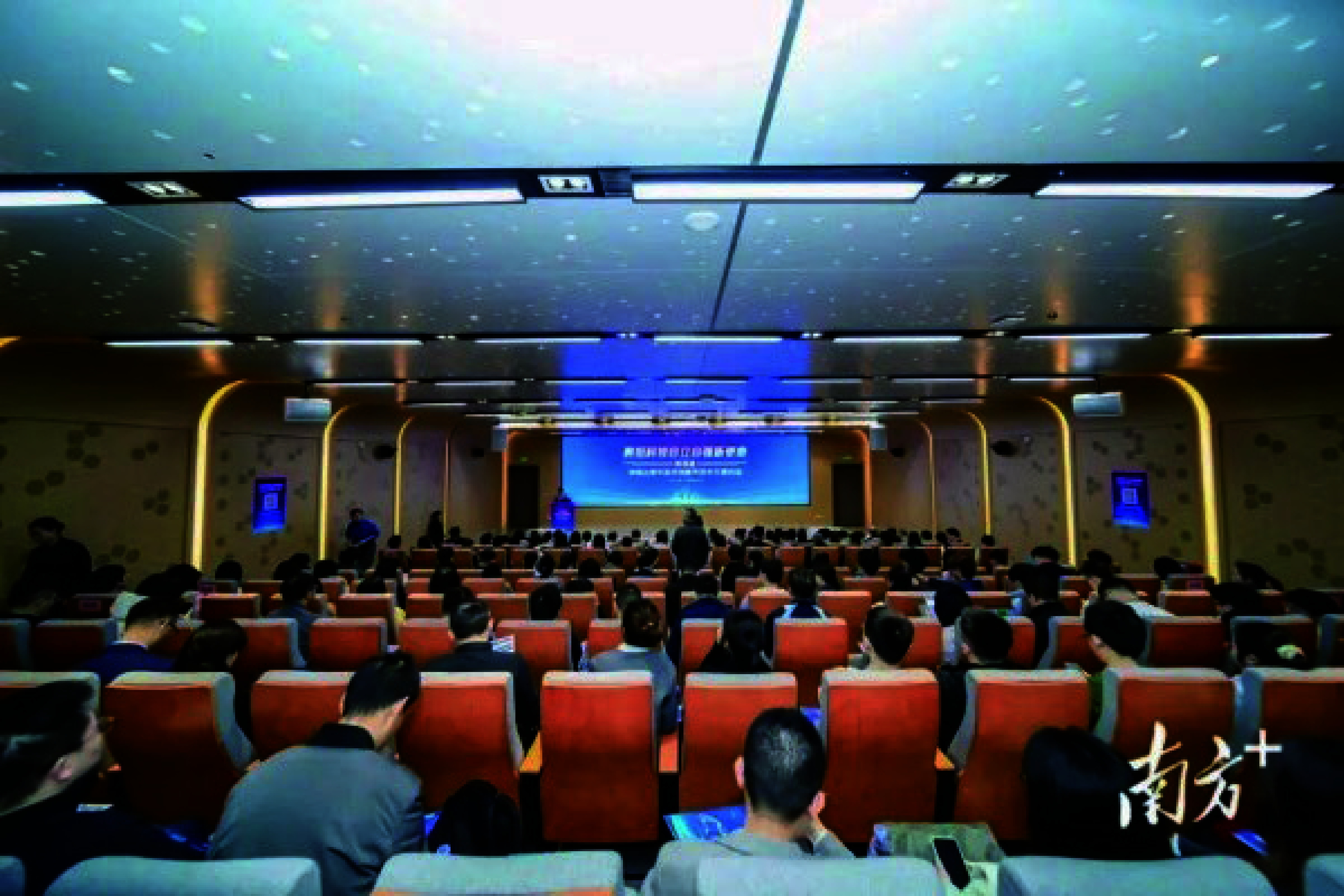# Zhao Yan wins Zhong Nanshan Youth Science and Technology Innovation Award

**DOI:** 10.52601/bpr.2026.250904

**Published:** 2026-02-28

On December 28, 2025，the theme event for the announcement of the Fourth Zhong Nanshan Youth Science and Technology Innovation Award was held at the Guangzhou Laboratory.

During the event, the list of award winners was revealed, with Zhao Yan, secretary-general of the Molecular Biophysics Branch of the Biophysical Society of China, among the recipients.

**Figure 1:**
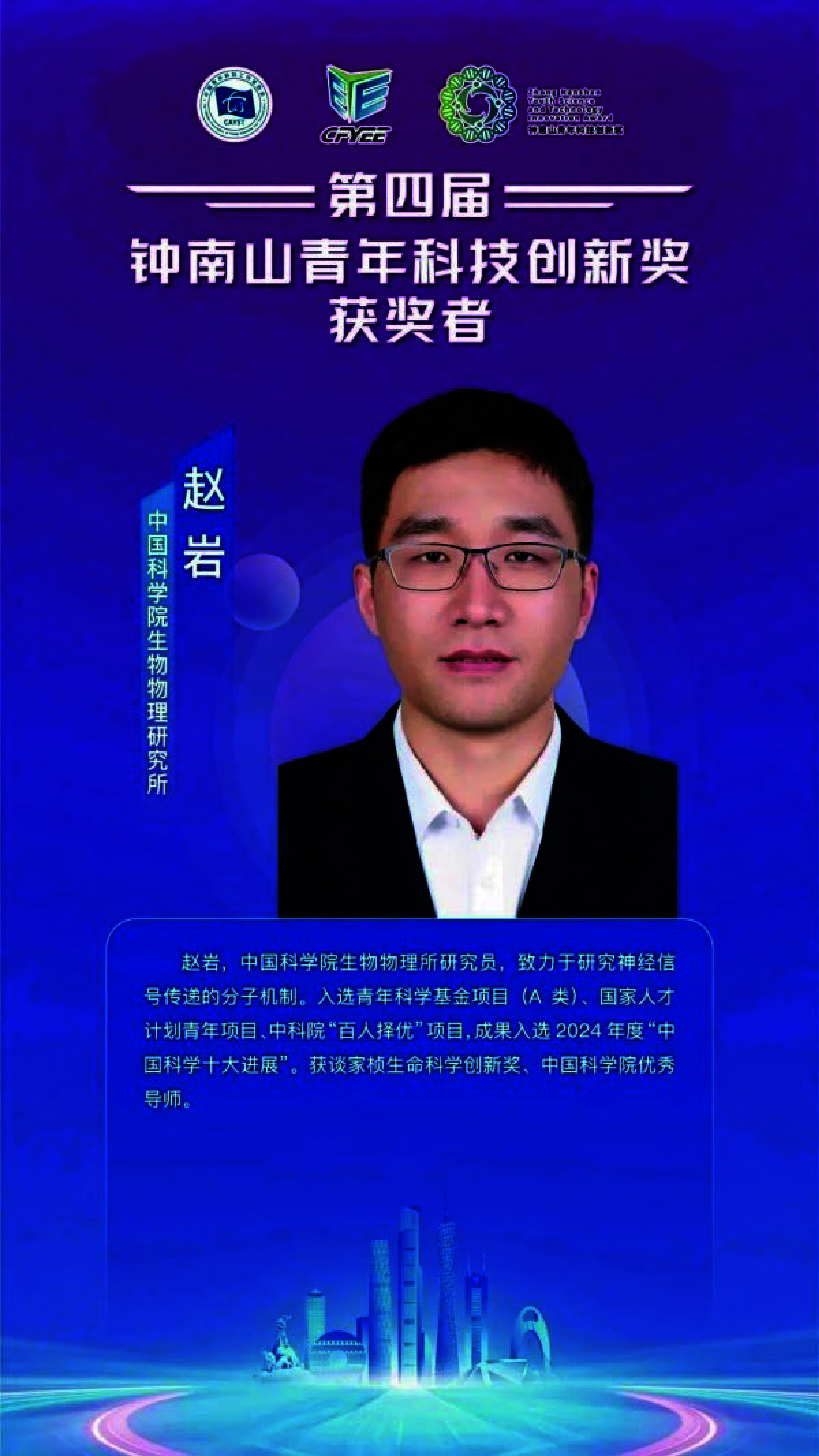


Zhao Yan, secretary-general of the Molecular Biophysics Branch of the Biophysical Society of China, wins Fourth Zhong Nanshan Youth Science and Technology Innovation Award.

Established in 2020, the Zhong Nanshan Youth Science and Technology Innovation Award is now in its fourth edition. The award, approved by the Secretariat of the Central Committee of the Communist Youth League and authorized by Academician Zhong Nanshan, aims to encourage young people to learn from Zhong Nanshan's patriotic spirit and sense of responsibility. It seeks to inspire them to continuously forge ahead, engage in innovative practices, and promote innovation and development in the fields of medical and health services and life sciences. Each year, the award is open to young scientific and technological workers under the age of 40 across the nation in the fields of medicine and life sciences, with no more than 10 recipients selected per edition.

**Figure 2:**